# P-1032. Posaconazole for Prevention of COVID-19 Associated Pulmonary Aspergillosis in Mechanically Ventilated Patients: the POSACOVID Study

**DOI:** 10.1093/ofid/ofae631.1222

**Published:** 2025-01-29

**Authors:** Juergen Prattes, Daniele R Giacobbe, Cristina Marelli, Alessio Signori, Silvia Dettori, Greta Cattardico, Jonas Frost, Florian Reizine, Matteo Bassetti, Jean-Pierre Gangneux, Martin Hoenigl

**Affiliations:** Medical University of Graz, Graz, Steiermark, Austria; University of Genoa, Genoa, Liguria, Italy; IRCCS Ospedale Policlinico San Martino, Genoa, Liguria, Italy; University of Genoa, Genoa, Liguria, Italy; University of Genoa, Genoa, Liguria, Italy; University of Genoa, Genoa, Liguria, Italy; Medical University of Graz, Graz, Steiermark, Austria; University of Rennes, Rennes, Bretagne, France; Department of Health Science (DISSAL), Infectious Diseases Unit,, Genova, Liguria, Italy; University of Rennes, Rennes, Bretagne, France; Division of Infectious Diseases, Department of Internal Medicine, Medical University of Graz, Graz, Austria, Graz, Steiermark, Austria

## Abstract

**Background:**

The relative number of COVID-19 patients developing COVID-19 associated pulmonary aspergillosis (CAPA) has been increasing within the vaccination area and mortality is still high. This study investigated the impact of posaconazole (POSA) prophylaxis in COVID-19 patients with acute respiratory failure receiving corticosteroids on the risk for development of CAPA.

**Table 1:**
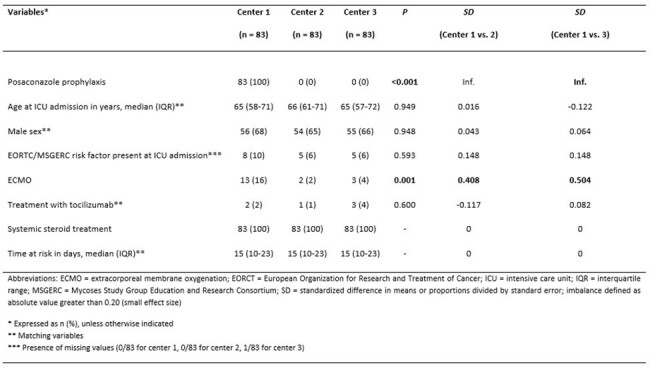
Demographic and clinical characteristics of patients after matching

**Methods:**

The primary aim of this prospective, multicenter, case-control study was to assess whether application of POSA prophylaxis in mechanically ventilated COVID-19 patients reduces the risk for CAPA development. All consecutive patients from center 1 (cases) who received POSA prophylaxis as standard-of-care were matched to one subject from center 2 and center 3 who did not receive any antifungal prophylaxis (Figure 1), using propensity score matching for the following variables: (i) age; (ii) sex; (iii) treatment with tocilizumab; (iv) time at risk.

**Figure 1:**
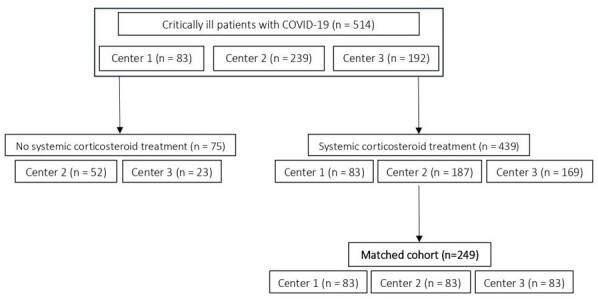
Matched cohorts after 1:1 and 1:1:1 matching. Both composed of 249 patients, although some different controls could have been selected.

**Results:**

Eighty-three consecutive patients receiving POSA were identified at center 1 and matched to 166 controls (Table 1). The pre-matching CAPA incidence rates were 1.69 CAPA/1000 ICU days in center 1, 1.42 CAPA/1000 ICU days in center 2 and 9.53 CAPA/1000 ICU days in center 3. The CAPA incidence rate ratio before matching was 2.38 (95% CI 0.87–9.08; p = 0.072) for those not receiving prophylaxis versus those who did. In post-matching multivariable logistic regression, presence of an EORTC/MSG risk factor at ICU admission (OR 4.35) and Center (Center 3 versus 1: OR 6.07; 95% CI 1.76 – 20.91; p = 0.004; Center 2 versus 1: not significant) were associated with CAPA development.

**Conclusion:**

The impact of POSA prophylaxis depends on the baseline CAPA incidence rate, which varies widely between centers and underlying individual patient risk factors. Future trials should therefore investigate targeted antifungal prophylaxis in COVID-19 patients.

**Disclosures:**

**Juergen Prattes, MD, PhD**, AbbVie Inc.: Stocks/Bonds (Private Company)|Gilead: Honoraria|MSD: Grant/Research Support|Novo Nordisk: Stocks/Bonds (Private Company)|Pfizer: Grant/Research Support|Pfizer: Honoraria **Daniele R. Giacobbe, M.D.**, bioMerieux: Grant/Research Support|Gilead Italia: Grant/Research Support|Menarini: Honoraria|Pfizer: Grant/Research Support|Pfizer: Honoraria|Shionogi: Grant/Research Support|Tillotts Pharma: Advisor/Consultant **Matteo Bassetti, PhD**, Angelini: Advisor/Consultant|Angelini: Honoraria|Astellas: Advisor/Consultant|Astellas: Honoraria|bioMerieux: Advisor/Consultant|bioMerieux: Honoraria|Cidara: Advisor/Consultant|Cidara: Honoraria|Gilead: Advisor/Consultant|Gilead: Honoraria|Menarini: Advisor/Consultant|Menarini: Honoraria|MSD: Advisor/Consultant|MSD: Honoraria|Nabriva: Advisor/Consultant|Nabriva: Honoraria|Pfizer: Advisor/Consultant|Pfizer: Honoraria|Tetraphase: Advisor/Consultant|Tetraphase: Honoraria **Jean-Pierre Gangneux, Prof.**, Gilead: Honoraria|MundiPharma: Advisor/Consultant|MundiPharma: Honoraria|Pfizer: Honoraria|Shionogi: Honoraria **Martin Hoenigl, MD**, Aicuris: Advisor/Consultant|Astra Zeneca: Honoraria|Gilead: Grant/Research Support|Gilead: Honoraria|IMMY: Grant/Research Support|Melinta: Grant/Research Support|Melinta: Honoraria|MSD: Grant/Research Support|Mundipharma: Grant/Research Support|Mundipharma: Honoraria|Pfizer: Grant/Research Support|Pulmocide: Advisor/Consultant|Pulmocide: Grant/Research Support|Scynexis: Advisor/Consultant|Scynexis: Grant/Research Support|Shionogi: Honoraria

